# Author Correction: Room-temperature valley coherence in a polaritonic system

**DOI:** 10.1038/s41467-019-12447-4

**Published:** 2019-09-27

**Authors:** L. Qiu, C. Chakraborty, S. Dhara, A. N. Vamivakas

**Affiliations:** 1 The Institute of Optics, University of Rochester, Rochester, NY 14627 USA; 2 Center for Coherence & Quantum Optics, University of Rochester, Rochester, NY 14627 USA; 3 Materials Science, University of Rochester, Rochester, NY 14627 USA; 4 Department of Physics, Indian Institute of Technology, Kharagpur, 721302 India

**Keywords:** Nanoscale devices, Optical materials and structures

Correction to: *Nature Communications* 10.1038/s41467-019-09490-6, published online 03 April 2019.

The original version of this Article omitted the following from the end of the Acknowledgements: ‘This work was supported by NSF EAGER: 1836566, NSF EFRI EFMA-1542707, NSF CAREER DMR 1553788, AFOSR FA9550-19-1-0074, the Cornell Center for Materials Research with funding from the NSF MRSEC program (DMR- 1719875) and the University of Rochester University Research Award and the Leonard Mandel Faculty Fellowship in Quantum Optics’. This has now been corrected in both the PDF and HTML versions of the Article.

