# Supplementary material for: Room-temperature valley coherence in a polaritonic system
Source: Nat Commun. 2019 Apr 3;10:1513. doi: 10.1038/s41467-019-09490-6 (PMC6447535; doi:10.1038/s41467-019-09490-6)
Supplement: Supplementary file 1 — Supplementary Information [file 41467_2019_9490_MOESM1_ESM.pdf]

Room-Temperature Valley Coherence in a Polaritonic System

Qiu et al.

SUPPLEMENTARY NOTE 1: MEASUREMENT APPARATUS

Supplementary Figure 1a shows the setup for angle-resolved polariton photoluminescence (PL). The excitation laser is reflected by a beam splitter into a 0.7 NA objective, which focuses the laser onto the sample. The PL is collected by the same objective, providing a wide range for collection angle θ between $\pm 44.4°$. Due to the clipping of the optics, the effective range for collecting angular information is $\pm 24°$. At the back focal plane (Fourier plane) of the objective, each point corresponds to a specific spatial frequency. A tube lens relays the Fourier plane to the spectrometer entrance slit. The Fourier plane (FP) and its conjugate (FP') are highlighted by green. The angle- and energy-resolved intensity map, as shown in Supplementary Figure 1b, can be directly obtained from the spectrometer CCD camera. The vertical linecut (Supplementary Figure 1c), representing the spectrum of a specific output angle, is fitted to get the polariton dispersion curve. Polarizers and wave plates are introduced for polarization-resolved measurement. The 752nm longpass is used to block the input laser, which will simultaneously cut off the information of the upper polariton.

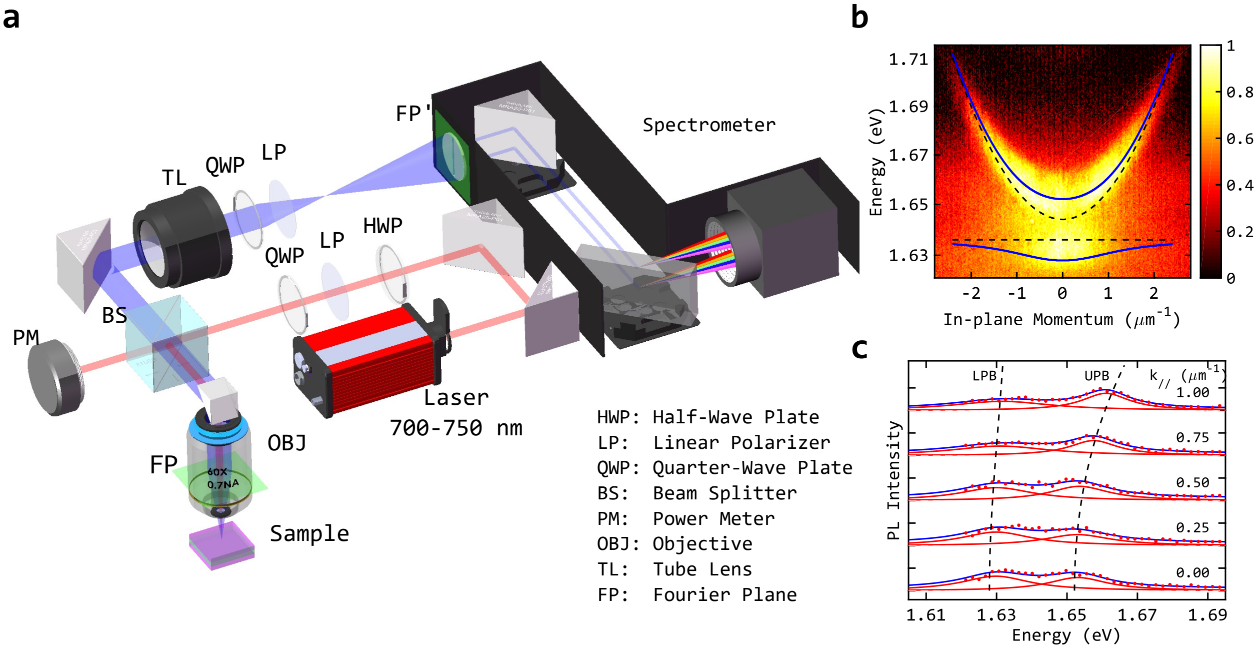

Supplementary Figure 1. **Fourier-plane spectroscopy.** **a,** System setup. Optical element schematics in use are from Thorlabs. **b,** Angle-resolved PL. The horizontal and vertical axis represent incident angle (in-plane momentum) and emission energy. Solid lines are fitted to a coupled-harmonic oscillator model. Dispersion relations of the uncoupled cavity photon(top) and exciton (bottom) are marked by dashed lines. **c,** Vertical linecuts of the PL map. Red points represent the spectrum of a specific in-plane momentum. The data are fitted by a double-lorentzian function (blue curve). Red lines stand for the two polariton branches. Dashed lines are a guide for the polariton dispersion.

The system is pump by single mode laser above the exciton energy gap. Tthermalization process populate the lower-polariton brach after excitation, as shown in Supplementary Figure 2. PLE data is obtained by sweeping the excitation laser from 1.658 eV to 1.774 eV.

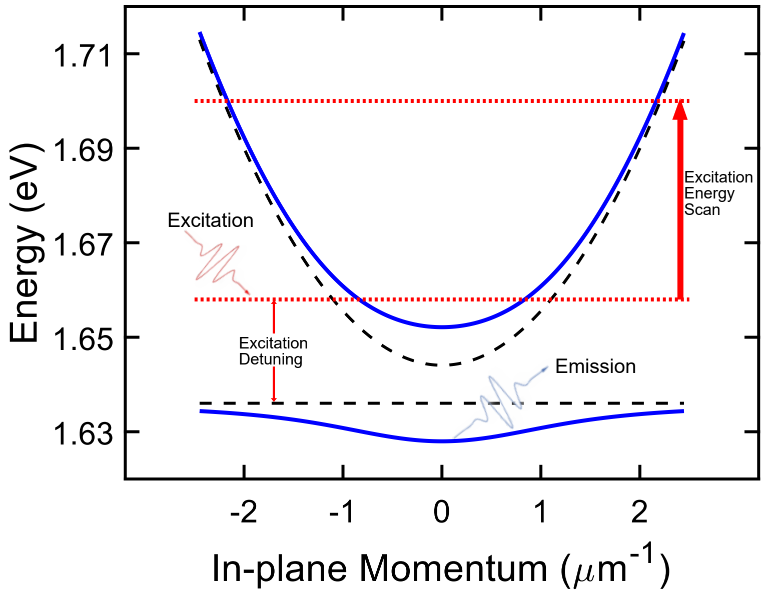

Supplementary Figure 2. **Laser excitation schematic.** The black(dashed) curves show the dispersion of uncoupled cavity mode and exciton. The blue(solid) curves indicate the polariton branches. The excitation laser energy is illustrated by the red(dotted) curves. The excitation detuning, ΔE is also defined in the illustration.

SUPPLEMENTARY NOTE 2: THEORETICAL MODEL

In order to study the polariton valley-related behavior, we build a valley-specific Jaynes-Cummings model. The model consisits of two valley-resolved exciton levels (K and K' valley) and two helicity-resolved cavity modes (RHCP and LHCP cavity)[1]. Coupling of the cavity photon and exciton results in coherent transitions between K/K' valley and RHCP/LHCP cavity, marked by "Interaction". These four states are connected to the ground state by their individual population decay ("Decay"). We include both population transition ("Intervalley Scattering") and pure dephasing ("Dephasing") between the two valleys to account for valley-polariton depolarization and valley-polariton decoherence and depolarization. Dephasing of the two cavity modes is neglected due to the small TM/TE mode splitting. Additionally, the incoherent pump only excites the two valley exciton states.

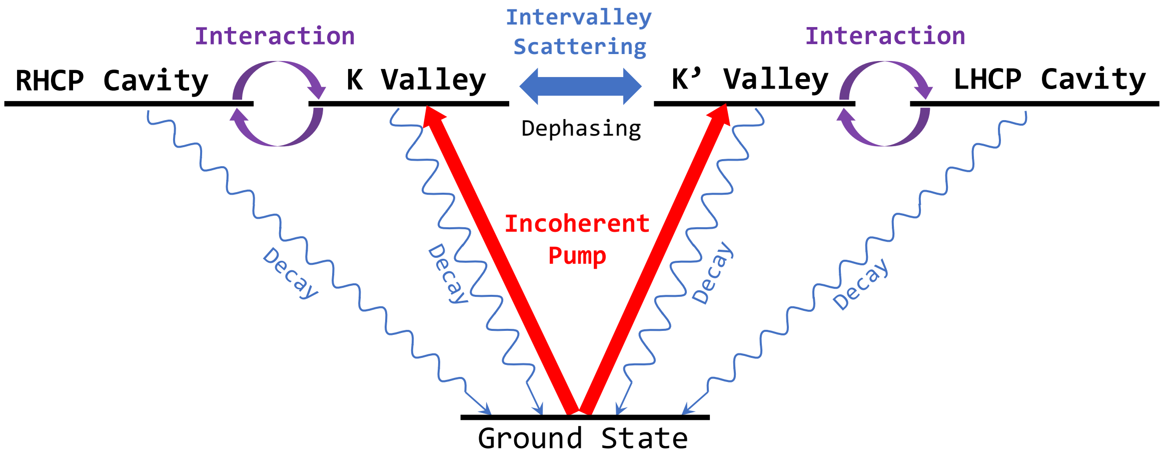

Supplementary Figure 3. **Illustration of the valley-specific Jaynes-Cummings model.**

We use a master-equation approach to solve for the steady-state solution of the system's density matrix[2]

$$\frac{d\rho}{dt} = \mathcal{L}\rho. \tag{1}$$

where $\mathcal{L}$ is the Liouville operator which governs the time evolution of the density matrix. The first term of the Liouville is the interaction directly from the system Hamiltonian, which illustrates the strong coupling between the K (K') valley exciton and the RHCP (LHCP) cavity mode

$$(\mathcal{L}\rho)_{\text{interaction}} = -i[H, \rho]. \tag{2}$$

Population decay can be expressed as following.

$$(\mathcal{L}\rho)_{\text{decay}} = -\frac{\gamma_{\text{i}}}{2}(\sigma_{\text{i}}^{+}\sigma_{\text{i}}^{-}\rho + \rho\sigma_{\text{i}}^{+}\sigma_{\text{i}}^{-} - 2\sigma_{\text{i}}^{+}\rho\sigma_{\text{i}}^{-})|_{\text{i=A,B,a,b}} \tag{3}$$

where A and B represent the helicity-resolved cavity modes (RHCP and LHCP cavity) and a and b stand for the corresponding valley exciton modes (K and K' valley). γ_{i} is a phenomenological constant which captures the population decay rate.

The incoherent pump term can be separated into two parts. The first part is the pump into a specific valley

$$(\mathcal{L}\rho)_{\text{pump1}} = -\frac{R_{\text{j}}}{2}(\sigma_{\text{j}}^{+}\sigma_{\text{j}}^{-}\rho + \rho\sigma_{\text{j}}^{+}\sigma_{\text{j}}^{-} - 2(\sigma_{\text{j}}^{+}\rho\sigma_{\text{j}}^{-} + \sigma_{\text{j}}^{-}\rho\sigma_{\text{j}}^{+}))|_{\text{j=a,b}}. \tag{4}$$

The second part is the simultaneous pumping of the two valleys[3] that will be prominent when exciting the system with a linearly polarized laser. A superposition of K and K' valley is created and the master equation form of this pump process can be written as

$$(\mathcal{L}\rho)_{\text{pump2}} = -\frac{R_{\text{ab}}}{2}((\sigma_{\text{a}}^{+}\sigma_{\text{b}}^{-}+\sigma_{\text{b}}^{+}\sigma_{\text{a}}^{-})\rho+\rho(\sigma_{\text{a}}^{+}\sigma_{\text{b}}^{-}+\sigma_{\text{b}}^{+}\sigma_{\text{a}}^{-})-2(\sigma_{\text{a}}^{+}\rho\sigma_{\text{b}}^{-}+\sigma_{\text{b}}^{+}\rho\sigma_{\text{a}}^{-}+\sigma_{\text{a}}^{-}\rho\sigma_{\text{b}}^{+}+\sigma_{\text{b}}^{-}\rho\sigma_{\text{a}}^{+})) \tag{5}$$

where $R_{\text{ab}} = \sqrt{R_{\text{a}}R_{\text{b}}}$. The valley population transition induced by scattering can be expressed as,

$$(\mathcal{L}\rho)_{\text{scatter}} = -\frac{\gamma_{\text{v}}}{2}((\sigma_{\text{a}}^{+}\sigma_{\text{a}}^{-}\rho+\rho\sigma_{\text{a}}^{+}\sigma_{\text{a}}^{-}-2\sigma_{\text{b}}^{+}\sigma_{\text{a}}^{-}\rho\sigma_{\text{a}}^{+}\sigma_{\text{b}}^{-})+(\sigma_{\text{b}}^{+}\sigma_{\text{b}}^{-}\rho+\rho\sigma_{\text{b}}^{+}\sigma_{\text{b}}^{-}-2\sigma_{\text{a}}^{+}\sigma_{\text{b}}^{-}\rho\sigma_{\text{b}}^{+}\sigma_{\text{a}}^{-})) \tag{6}$$

Aside from the above, the density matrix dynamics will also be affected by pure dephasing processes [shown in Supplementary Equation 7]

$$(\mathcal{L}\rho)_{\text{dephase}} = -\frac{\gamma_{\text{dep}}}{2}((\sigma_{\text{a}}^{+}\sigma_{\text{a}}^{-}-\sigma_{\text{b}}^{+}\sigma_{\text{b}}^{-})\rho(\sigma_{\text{a}}^{+}\sigma_{\text{a}}^{-}-\sigma_{\text{b}}^{+}\sigma_{\text{b}}^{-})-(\sigma_{\text{a}}^{+}\sigma_{\text{a}}^{-}+\sigma_{\text{b}}^{+}\sigma_{\text{b}}^{-})\rho(\sigma_{\text{a}}^{+}\sigma_{\text{a}}^{-}+\sigma_{\text{b}}^{+}\sigma_{\text{b}}^{-})) \tag{7}$$

where γ_{v} and γ_{dep} account for the intervalley scattering rate and the pure dephasing rate. The full master equation is a summation of all the separate terms above. The steady-state solution is obtained by setting the time derivative to zero. Individual density matrix element can be calculated from the 16 coupled differential equations.
For the steady-state valley-polariton coherence ρ_{l}, R_{a} and R_{b} are set equal to each other. The creation operator of the lower polariton can be expressed as,

$$P_{\text{K(K')}}^{+} = X\sigma_{\text{a(b)}}^{+} + C\sigma_{\text{A(B)}}^{+} \tag{8}$$

Where X and C are the Hopfield's coefficient for the polariton's excitonic and photonic components. The population and coherence of the two valley polariton states can be expressed in the form of the density matrix elements in the exciton-cavity basis as

$$\begin{aligned} \rho_{P_{\text{K}}P_{\text{K}}} &= |X|^2\rho_{\text{aa}} + |C|^2\rho_{\text{AA}} + XC^*\rho_{\text{aA}} + X^*C\rho_{\text{Aa}} \\ \rho_{P_{\text{K}}P_{\text{K'}}} &= |X|^2\rho_{\text{ab}} + |C|^2\rho_{\text{AB}} + XC^*\rho_{\text{aB}} + X^*C\rho_{\text{Ab}}. \end{aligned} \tag{9}$$

The steady-state DOLP of the emission is expressed as

$$\rho_{\text{l}} = \frac{\rho_{P_{\text{K}}P_{\text{K'}}} + \rho_{P_{\text{K'}}P_{\text{K}}}}{\rho_{P_{\text{K}}P_{\text{K}}} + \rho_{P_{\text{K'}}P_{\text{K'}}}} = \frac{|X|^2(\rho_{\text{ab}} + \rho_{\text{ba}}) + |C|^2(\rho_{\text{AB}} + \rho_{\text{BA}}) + XC^*(\rho_{\text{aB}} + \rho_{\text{bA}}) + X^*C(\rho_{\text{Ab}} + \rho_{\text{Ba}})}{|X|^2(\rho_{\text{aa}} + \rho_{\text{bb}}) + |C|^2(\rho_{\text{AA}} + \rho_{\text{BB}}) + XC^*(\rho_{\text{aA}} + \rho_{\text{bB}}) + X^*C(\rho_{\text{Aa}} + \rho_{\text{Bb}})}. \tag{10}$$

According to the calculation, Supplementary Equation 10 can be reduced to Supplementary Equation 11, independent of the Hopfield coefficients,

$$\rho_{\text{l}} = \frac{\rho_{\text{ab}} + \rho_{\text{ba}}}{\rho_{\text{aa}} + \rho_{\text{bb}}} = \frac{\rho_{\text{AB}} + \rho_{\text{BA}}}{\rho_{\text{AA}} + \rho_{\text{BB}}} \tag{11}$$

We proceed the derivation in the weak pump limit, $R_{\mathrm{x}} \rightarrow 0$ (x = a, b),

$$\rho_{\mathrm{l}} = \frac{\gamma_{\mathrm{c}}\gamma_{\mathrm{x}}((\gamma_{\mathrm{c}}+\gamma_{\mathrm{v}}+\gamma_{\mathrm{x}})^2+4\delta\omega^2)+(\gamma_{\mathrm{c}}+\gamma_{\mathrm{x}})(\gamma_{\mathrm{c}}+\gamma_{\mathrm{v}}+\gamma_{\mathrm{x}})\Omega_{\mathrm{Rabi}}^2}{\gamma_{\mathrm{c}}(\gamma_{\mathrm{dep}}+\gamma_{\mathrm{v}}+\gamma_{\mathrm{x}})((\gamma_{\mathrm{c}}+\gamma_{\mathrm{v}}+\gamma_{\mathrm{x}})^2+4\delta\omega^2)+(\gamma_{\mathrm{c}}+\gamma_{\mathrm{dep}}+\gamma_{\mathrm{v}}+\gamma_{\mathrm{x}})(\gamma_{\mathrm{c}}+\gamma_{\mathrm{v}}+\gamma_{\mathrm{x}})\Omega_{\mathrm{Rabi}}^2} \tag{12}$$

where Ω_{Rabi} is the Rabi frequency between the cavity photon and exciton, $\delta\omega$ is the cavity-exciton energy detuning. Supplementary Equation 12 will reduce to Eq. (2) in the main text by assuming in the strong coupling regime, $\Omega_{\mathrm{Rabi}} \gg \gamma_{\mathrm{i}}$ (i = c,v,x,dep), $\delta\omega$.

$$\rho_{\mathrm{l}} = \frac{1}{1+\frac{\gamma_{\mathrm{dep}}+\gamma_{\mathrm{v}}}{\gamma_{\mathrm{c}}+\gamma_{\mathrm{x}}}} \tag{13}$$

Eq. (3) in the main text can be recovered by the assumption $\Omega_{\mathrm{Rabi}} = 0$

$$\rho_{\mathrm{l}} = \frac{1}{1+\frac{\gamma_{\mathrm{dep}}+\gamma_{\mathrm{v}}}{\gamma_{\mathrm{x}}}}. \tag{14}$$

To study the steady-state valley-polariton helicity ρ_{c} (c identifies circular) one of the pump channels is set to zero ($R_b = 0$). The degree of circular polarization can be expressed as the relative population difference between two valleys

$$\rho_{\mathrm{c}} = \frac{\rho_{\mathrm{aa}}-\rho_{\mathrm{bb}}}{\rho_{\mathrm{aa}}+\rho_{\mathrm{bb}}}. \tag{15}$$

In the weak-pump approximation,

$$\rho_{\mathrm{c}} = \frac{\gamma_{\mathrm{c}}\gamma_{\mathrm{x}}((\gamma_{\mathrm{c}}+\gamma_{\mathrm{v}}+\gamma_{\mathrm{x}})^2+4\delta\omega^2)+(\gamma_{\mathrm{c}}+\gamma_{\mathrm{x}})(\gamma_{\mathrm{c}}+\gamma_{\mathrm{v}}+\gamma_{\mathrm{x}})\Omega_{\mathrm{Rabi}}^2}{\gamma_{\mathrm{c}}(2\gamma_{\mathrm{v}}+\gamma_{\mathrm{x}})((\gamma_{\mathrm{c}}+\gamma_{\mathrm{v}}+\gamma_{\mathrm{x}})^2+4\delta\omega^2)+(\gamma_{\mathrm{c}}+2\gamma_{\mathrm{v}}+\gamma_{\mathrm{x}})(\gamma_{\mathrm{c}}+\gamma_{\mathrm{v}}+\gamma_{\mathrm{x}})\Omega_{\mathrm{Rabi}}^2} \tag{16}$$

The steady-state valley-polariton helicity reduces to the following form by assuming strong coupling

$$\rho_{\mathrm{c}} = \frac{1}{1+\frac{2\gamma_{\mathrm{v}}}{\gamma_{\mathrm{c}}+\gamma_{\mathrm{x}}}} \tag{17}$$

The uncoupled exciton's valley helicity can also be derived from Supplementary Equation 16

$$\rho_{\mathrm{c}} = \frac{1}{1+\frac{2\gamma_{\mathrm{v}}}{\gamma_{\mathrm{x}}}}. \tag{18}$$

In general, γ_{c} in the denominator is the only difference between the coupled and uncoupled polarization contrast. The linewidth of the DBR cavity ($\hbar\gamma_{\mathrm{c}}$) is measured as 6.85 meV. $\hbar\gamma_{\mathrm{x}} = 0.23$ meV is taken from the time-resolved PL intensity measurement with T1 = 18 ps[4]. Fast population decay dominated by photonic component ($\gamma_{\mathrm{c}} \gg \gamma_{\mathrm{x}}$) enables quick readout of valley information, which yields better polarization contrast.

SUPPLEMENTARY NOTE 3: TRANSFER MATRIX CALCULATION

The transfer matrix method (TMM) has been used to design resonant cavities with desired frequencies. In order to enhance light-matter interaction, the cavity resonance should be close to the WSe_2 exciton energy (∼1.65 eV at room temperature). As shown in Supplementary Figure 4a and 4b, with optimized parameters, the calculated cavity TM and TE resonances center around 1.65 eV for the case of normal incidence.

TM/TE mode splitting plays an important role in the polariton valley behaviors. As is

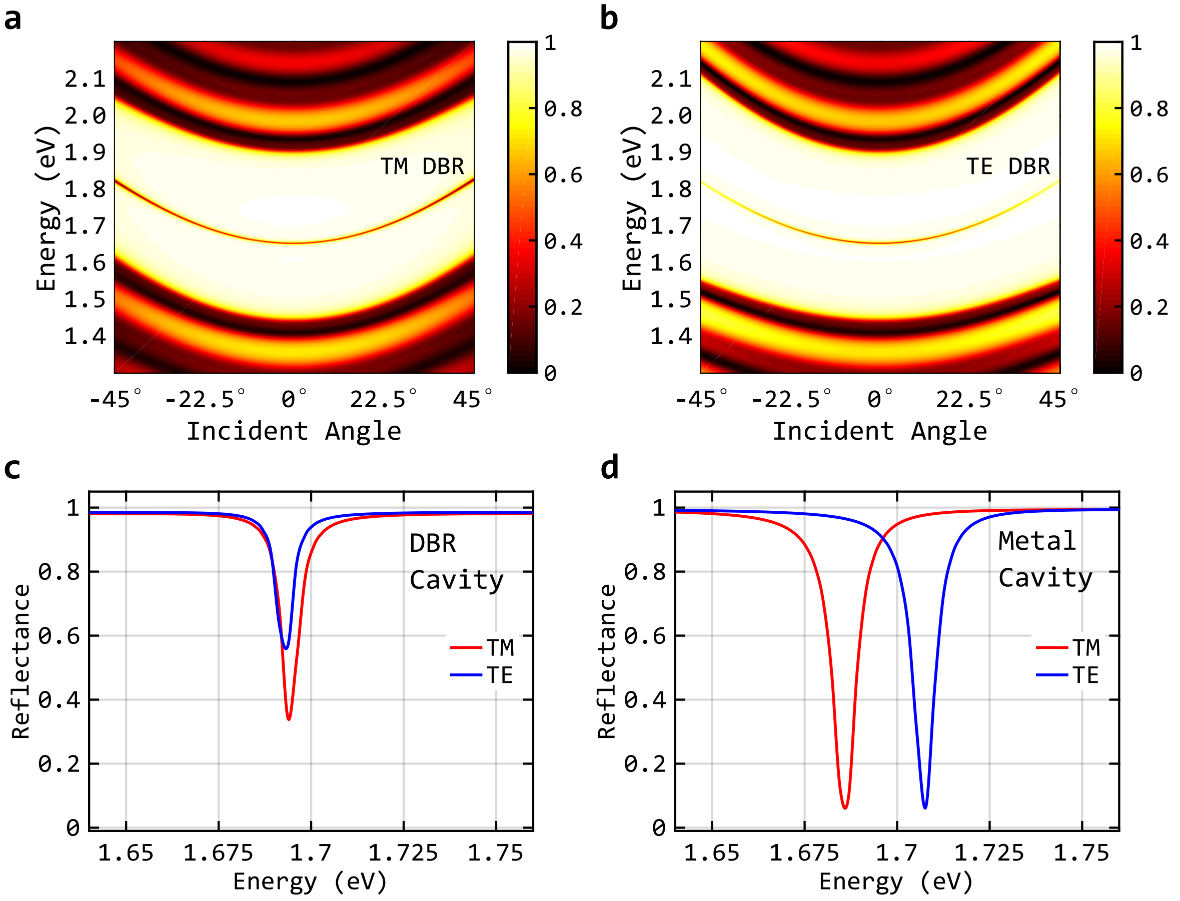

Supplementary Figure 4. **Cavity reflectance. a,b,** Angle-resolved reflectance for TM (**a**) and TE (**b**) cavity modes. **c,d,** TM/TE mode splitting for oblique incidence at 22.5°. DBR cavity (**c**) is composed of a $\lambda/2$ SiO_2 cavity layer encapsulated in two SiO_2/Ta_2O_5 bragg mirrors. Metal cavity (**d**) features a Ag-SiO_2-Ag structure.

well-known, the electron-hole interaction generates an effective magnetic field for exciton valley pseudospin, which results in valley decoherence. The photonic counterpart for this interaction is the TM/TE splitting, which will, equivalently, disturb the phase relation between the two modes. It is shown in Supplementary Figure 4c and 4d that, at oblique incidence (22.5°), cavities made of DBR and of Ag mirror feature distinct splittings even if they share the same resonance frequency at normal incidence. The TM/TE splitting of metal cavity (21.577 meV) is over one order of magnitude larger than that of dielectric cavity (1.344 meV). As a result, polaritons formed by dielectric cavity are more immune to photonic dephasing.

SUPPLEMENTARY NOTE 4: POLARTION VALLEY HELICITY

Analysis of the valley helicity

As predicted from the model, the steady-state valley-polariton valley helicity will be enhanced by coupling with the cavity mode. In order to prove its validity, we perform polarization-resolved PL measurements in the circularly polarized basis. Right-/Left-handed circularly polarized light (σ^+/σ^-) generates polariton states in the K/K' valley. The results are shown in Supplementary Figure 5c. As the excitation energy is sweeps from far above the WSe_2 bandgap (1.774 eV) to resonance (1.658 eV), ρ_c rises from 3% to 18% and saturates as the excitation approaches the WSe_2 exciton energy. The co-polarized intensity versus the cross-polarized intensity pumped at 1.664 eV (745 nm) is shown in Supplementary Figure 5a. For comparison, Supplementary Figure 5d shows the polarized-resolved PLE for the uncoupled sample. Even excited by a near-resonance pump, the uncoupled exciton shows negligible valley helicity (Supplementary Figure 5b).

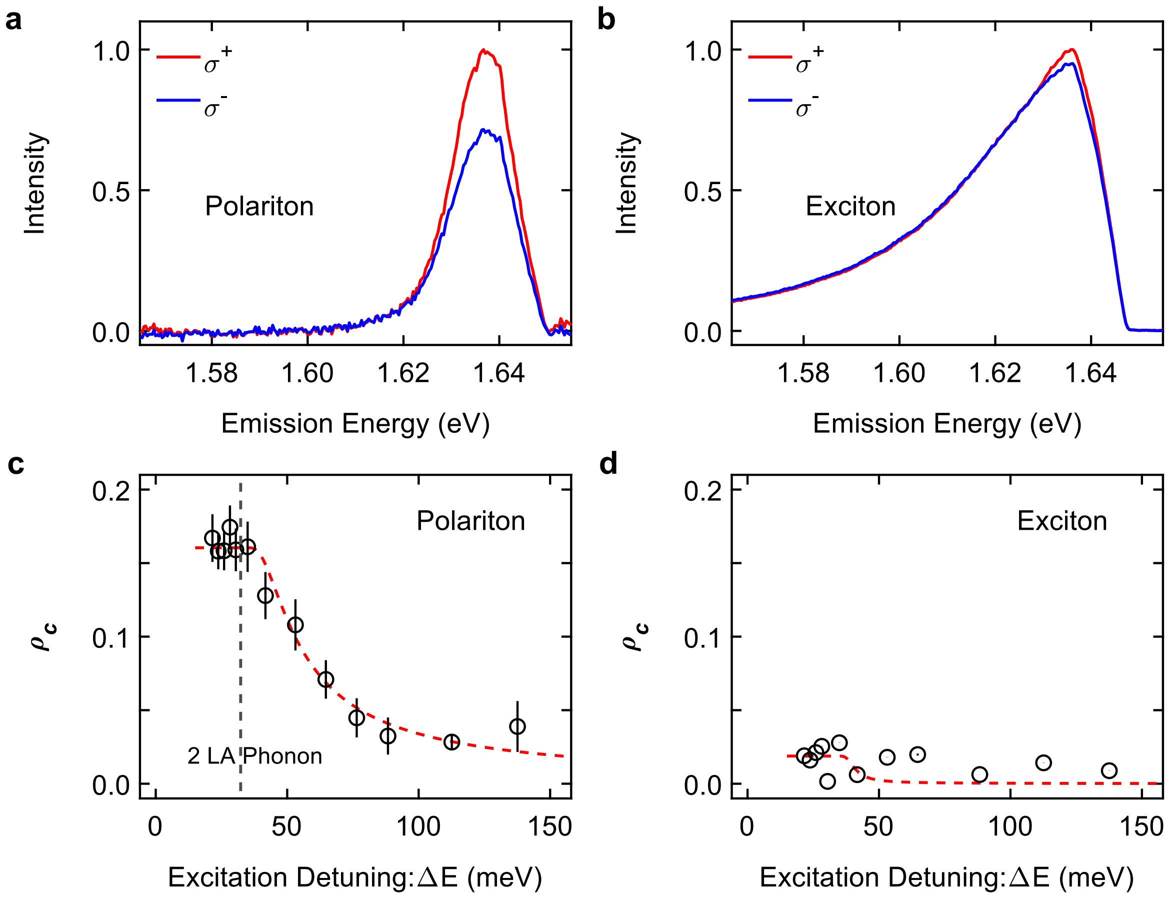

Supplementary Figure 5. **Valley helicity of polariton vs. bare exciton. a,b,** PL spectrum of lower polariton (**a**) and bare exciton (**b**) under 745nm σ^+ pump. Blue and red curves are detected at σ^+ and σ^-. **c,d,** Lower polariton (**c**) and bare exciton (**d**) valley helicity ρ_c as a function of excitation energy. Dashed lines are fitted by the valley-specific Jaynes-Cummings model. Errorbars represent the standard deviation among measurements.

According to Supplementary Equation 17 and 18, the energy dependence of valley helicity contrast $\rho_c(E)$ can be determined from the energy-modulation of the intervalley transition rate $\gamma_v(E)$. As discussed in the main text, the intervalley transition rate is dominated by phonon scattering and the rate is directly related to energy of the LA phonon. We can fit the energy-dependent curve in Supplementary Figure 5c and 5d by,

$$\rho_c = \frac{\rho_{c0}}{1 + A/(e^{\frac{\hbar\omega_q}{\Delta E - \hbar\omega_q}} - 1)} \tag{19}$$

ΔE represents the excitation detuning, while $\hbar\omega_q$ is the phonon energy involved in the process. The fitted LA phonon energy $\hbar\omega_q$ is two times the LA phonon energy of 16.15 meV (close to the theoretical prediction of $\sim$ 16 meV) as expected for the 2-phonon mediated process. In addition, with regard to the A parameter, $A_{\text{exciton}} \gg A_{\text{polariton}}$. Correspondingly, the cavity decay rate γ_c is much faster than the exciton decay rate γ_x.

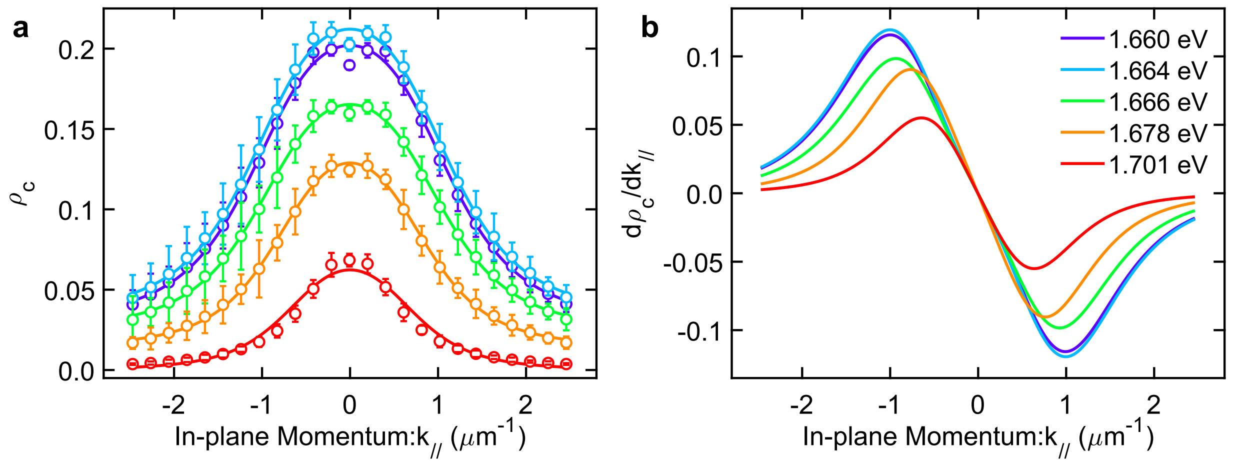

Supplementary Figure 6. **Angular dependence of polariton valley helicity. a,** Angle-resolved valley helicity ρ_c at different pump energies. Solid lines are fitting results. **b,** Derivatives of the corresponding polarization contrast. Errorbars represent the standard deviation among measurements.

Due to the strong dispersive cavity mode, the steady-state valley-polariton helicity will also be dependent on angle. It is shown in Supplementary Figure 6a that ρ_c drops as a function of in-plane momentum. Also, as the excitation is tuned away from the exciton energy, the overall contrast level decreases. This is consistent with the behavior of valley coherence in Fig. 4 of main text. The angle dependent data can be fitted with Supplementary Equation 16 as the solid lines in Supplementary Figure 6a. Derivatives of valley polarization (Supplementary Figure 6b) further validate the influence of excitation, where large excessive energy tends to reduce the angular dependence of ρ_c.

Role of pure-dephasing in steady-state valley-polariton coherence

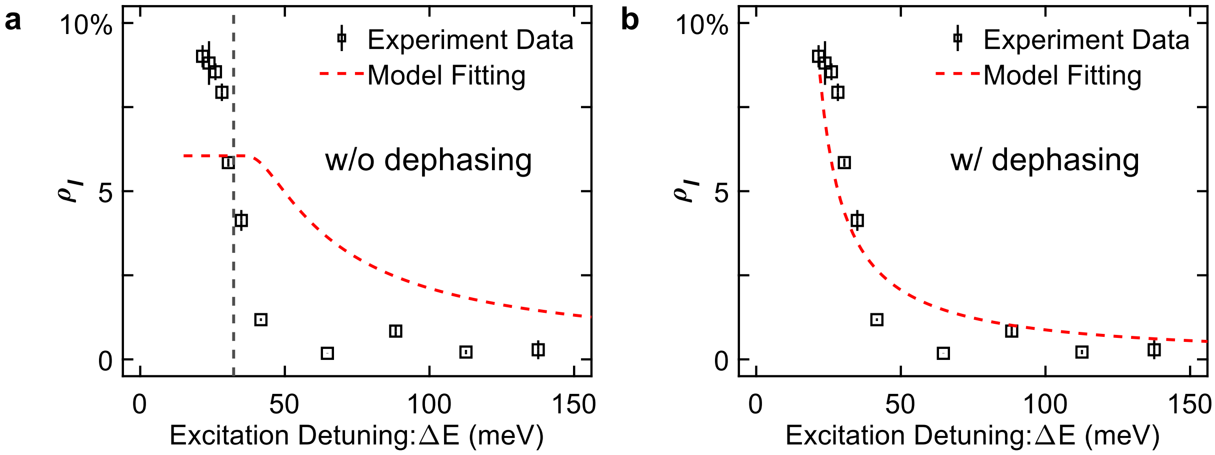

Supplementary Figure 7. **Energy dependence of steady-state valley-polariton coherence. a,b,** Comparsion of fittings without (a) and with (b) intervalley pure dephasing. Errorbars represent the standard deviation among measurements.

Despite describing different aspects of valley dynamics, ρ_c and ρ_l share many similarities. In Supplementary Equation 13 and 17, both contrasts depend on the intervalley scattering rate. In fact, if neglecting the pure dephasing rate, ρ_l and ρ_c are only differentiated by a factor of 2 in the denominator. However, the fitting function (without dephasing) cannot fit well with the PLE result of ρ_l, as shown in Supplementary Figure 7a. Alternatively, if we consider the pure dephasing rate induced by the MSS mechanism, the fitting is in accordance with the data (Supplementary Figure 7b and Fig. 3c in the main text).

SUPPLEMENTARY REFERENCES

[1] Chen, Y.-J., Cain, J. D., Stanev, T. K., Dravid, V. P. & Stern, N. P. Valley-polarized exciton-polaritons in a monolayer semiconductor. *Nature Photonics* **11**, 431–435 (2017).

[2] Scully, M. O. & Zubairy, M. S. Quantum optics (1999).

[3] Kozlov, V. V., Rostovtsev, Y. & Scully, M. O. Inducing quantum coherence via decays and incoherent pumping with application to population trapping, lasing without inversion, and quenching of spontaneous emission. *Physical Review A* **74** (2006).

[4] Cui, Q., Ceballos, F., Kumar, N. & Zhao, H. Transient absorption microscopy of monolayer and bulk wse2. *ACS nano* **8**, 2970–2976 (2014).
